# Ubrogepant to Treat Acute Migraine in Adults

**DOI:** 10.3390/neurolint13010004

**Published:** 2021-01-28

**Authors:** Amber N. Edinoff, C. Austin Casey, Marc A. Colon, Alexa R. Zaheri, Courtney M. Gregoire, Margaret M. Bourg, Alan D. Kaye, Jessica S. Kaye, Adam M. Kaye, Rachel J. Kaye, Sridhar R. Tirumala, Omar Viswanath, Ivan Urits

**Affiliations:** 1 Department of Psychiatry and Behavioral Medicine, Louisiana State University Health Shreveport, Shreveport, LA 71103, USA; ccasey@lsuhsc.edu (C.A.C.); MColon@lsuhsc.edu (M.A.C.); 2 Shreveport School of Medicine, Louisiana State University, Shreveport, LA 71103, USA; azahe1@lsuhsc.edu (A.R.Z.); cgreg3@lsuhsc.edu (C.M.G.); mbour8@lsuhsc.edu (M.M.B.); 3 Department of Anesthesiology, Louisiana State University Shreveport, Shreveport, LA 71103, USA; akaye@lsuhsc.edu (A.D.K.); stirum@lsuhsc.edu (S.R.T.); viswanoy@gmail.com (O.V.); ivanurits@gmail.com (I.U.); 4 Department of Pharmacy Practice, Thomas J. Long School of Pharmacy and Health Sciences, University of the Pacific, Stockton, CA 95211, USA; j_kaye1@u.pacific.edu (J.S.K.); akaye@PACIFIC.EDU (A.M.K.); 5 School of medicine, Medical University of South Carolina, Charleston, SC 29425, USA; rachelkaye17@hotmail.com; 6 Department of Anesthesiology, University of Arizona College of Medicine-Phoenix, Phoenix, AZ 85004, USA; 7 Department of Anesthesiology, Creighton University School of Medicine, Omaha, NE 68124, USA; 8 Valley Anesthesiology and Pain Consultants—Envision Physician Services, Phoenix, AZ 85004, USA; 9 Southcoast Health, Southcoast Physicians Group Pain Medicine, Wareham, MA 02571, USA

**Keywords:** ubrogepant, migraines, migraine treatment, abortive treatment, CGRP inhibitors

## Abstract

Migraine is a neurobiological headache disorder that affects around 16% of adults in the United States. Medical treatment of mild to moderate migraines include non-prescription non-steroidal anti-inflammatory drugs, acetaminophen, or aspirin and caffeine-containing combination analgesics. Additionally, moderate to severe migraines and those that are mild to moderate that have not responded to analgesics can be treated with triptans, which are drugs specific for migraine treatment. Non-pharmacological treatments include cognitive behavioral therapy and relaxation training. Medications for the prevention of migraines have also been developed since they are more affective in offsetting the symptoms. Ubrogepant’s high specificity and selectivity for calcitonin gene-related peptide (CGRP) sets it apart from certain other drugs, which previously limited the treatment of migraines with or without aura due to their decreased selectivity. The most frequently reported side effects are oropharyngeal pain, nasopharyngitis, and headache. Most studies found that participants receiving Ubrogepant were free from pain within 2 h when compared to placebo. Patients taking Ubrogepant should avoid taking it when pregnant or with end stage renal disease. In summary, Ubrogepant has good tolerability and an overall favorable safety profile. It appears to hold promise for the acute treatment of migraines with or without aura in adults.

## 1. Introduction

Migraine is a neurobiological headache disorder that affects around 16% of adults in the United States [1,2,3]. It is a recurring and disabling condition that can cause a reduction in the quality of life including the ability to work or participate in social activities [1,4]. Migraine is caused by increased excitability of the central nervous system and is made up of four phases: the premonitory, aura, headache, and postdrome [1,5,6,7]. Sensory sensitivity is the greatest indicator of an individual in the premonitory phase which precedes a headache [5]. The aura phase may also precede a headache, but it only presents in around 30% of patients experiencing migraine [1,2,5]. An aura is caused by reactive vasodilation to cerebral vasoconstriction along with cortical blood flow reduction [1,8]. It can present with visual, hemisensory, and language abnormalities with visual being the most common [2,5]. The headache phase is triggered by activation of the trigeminovascular pathway which results in throbbing pain [5,6]. After the throbbing pain subsides, the patient may experience fatigue, cognitive difficulties, or mood change, which are features of the postdrome phase [5,6,7].

Patients experiencing migraine may be at a higher risk for certain psychiatric conditions. For instance, patients suffering migraine with aura are three times more likely to suffer from bipolar disorder compared to the general population [9,10,11,12]. Additionally, migraineurs are at a higher risk for sleep disorders and are 2.5 times more likely to suffer from depression [9,13,14,15]. Since stress can be a trigger for a migraine, abuse and post-traumatic stress disorder show higher prevalence among migraineurs [9,16]. Therefore, migraine can play a significant role in a patient’s mental health status. 

Migraines can also be classified as chronic in nature or can happen whenever a specific trigger is induced acutely. It can also be subdivided further into another type called vestibular migraine. About 2% of the general population experiences chronic migraines [17]. In order to diagnose a patient with this condition, he/she must suffer from at least 15 days of headaches for at least three consecutive months [17]. Some symptoms of chronic migraine include hypersensitivity to visual, auditory, and olfactory stimuli as well as nausea and vomiting [17,18]. Vestibular migraine is the most common cause of episodic vertigo [19,20]. Damage to the central vestibular and ocular motor systems may also be seen in patients with vestibular migraine [20]. Treatment is still adapting for these types of migraines as targets for them are different.

The newest class of medication approved to treat migraine in adults is known as calcitonin gene-related peptide (CGRP) antagonists [3]. Among these are ubrogepant and rimegepant, which inhibit CGRP at locations in the migraine pathway in order to block vasodilation and neurogenic inflammation [3,21]. Ubrogepant is prescribed to adult patients for acute migraine therapy with or without aura for acute, abortive treatment. However, it is not indicated as a preventative treatment [3,21,22].

## 2. Migraines

Migraines are a type of headache disorder that can be quite disabling [23]. These episodes can last for several hours or even days, and are impactful on a patient’s life. Many mechanisms have been suggested to explain the pathophysiology of this fairly common disorder. There are two major forms of migraines—with and without aura, with the latter being headaches with additional neurological symptoms [23]. Many risk factors have been associated with chronicity of migraines, most dealing with failure to effectively treat acute migraines [24].

### 2.1. Epidemiology

Around the world, approximately 1–2% of people are afflicted with chronic migraines [25]. Migraines affect 12% of the United States population, with some research studies stating as high a prevalence of 16% [1,2]. Prevalence of migraines in the United States varies by race. The highest prevalence being found among Caucasians while Asian Americans have been found to have the lowest prevalence of migraines [25]. In about 3% of patients, irregular episodes of migraines progress to chronic migraines. Females are more likely to be affected by chronic migraines than males [24]. Some families have even shown an autosomal dominant inheritance pattern of vestibular migraines [26,27,28].

### 2.2. Pathophysiology

While the exact mechanism of migraine pathophysiology is not completely understood, research utilizing functional imaging suggests it involves both structural and functional alterations in the brain [18]. One of the major areas is in the connecting circuits between the thalamus and the cortex, and this can be identified during and after migraine episodes [5]. Such changes have also been identified in the various phases of chronic migraine episodes. The premonitory phase shows electrophysiological changes. It has been suggested that these changes can manifest into processing of sensory information [5]. Structural and functional changes have also been identified in the brainstem [18]. 

Altered connections in various regions of the brain have also been identified as a potential source of migraines. These regions include the cortex, the thalamus, the hypothalamus, the brainstem, the amygdala, and the cerebellum [5]. Increased excitability has also been found in certain cortices involved in top-down processing [18]. This effect on top-down processing is associated with increased levels of oxidative stress and increased processing of nociceptive sensations. Therefore, these individuals are more sensitive to sensations that lead to migraine episodes [24]. 

Migraines can be related to molecular changes. For example, CGRP is secreted by trigeminal afferent nerves when the dura mater becomes inflamed. This release of CGRP propagates pain signals during a migraine episode. In fact, CGRP can serve as a biomarker in patients with chronic migraines [18]. Another molecule involved in the chronicity of migraines is 5-hydroxytryptamine (5-HT), which is involved in the modulation of pain and sleep pathways. Overuse of migraine medication and recurrent headaches can increase 5-HT, which results in serotonin receptor upregulation. This upregulation further enhances repeated episodes of headaches, eventually leading to chronic migraines [18]. Pituitary adenylate cyclase activating polypeptide (PACAP), also serves as a molecular influence on the pathophysiology. Frequent headaches lead to a decrease in PACAP and increase in its receptors which this is thought to serve a role in progression of migraines [18]. 

Drastic changes in the homeostasis of ions have been associated with visual aura. Frequent changes from depolarization to hyperpolarization leads to an imbalance of ions across cortical neurons, and large amounts of energy are needed to restore the balance. The increased blood flow associated with this increased demand for energy has been identified as a possible mechanism of visual aura [29]. 

### 2.3. Risk Factors 

Various risk factors of migraines have been identified. Women are at greater risk of having both chronic and more severe migraines than men [25]. The onset of migraines in women is roughly before or near puberty and lasts well into postmenopausal stages [30]. Those with chronic migraines are more likely of lower socioeconomic status [31]. Another risk factor is the overuse of acute migraine medication. With repetitive overuse of these medications, the frequency of migraine episodes increases [24]. The risk of having chronic migraines can also be doubled if acute migraines are treated ineffectively. Inadequately treating migraines can also cause sensitization due to longer periods of experiencing headaches [24]. Mood disorders have been associated with increased risk of chronic migraines [18]. Comorbidities of chronic migraines includes diseases associated with the respiratory, cardiovascular, and psychiatric disease. Specifically, chronic migraines are twice as likely to be associated with recurrent bronchitis, ulcers, depression, and other pain disorders [31].

### 2.4. Presentation

Patients experiencing migraines usually present with a combination of severe headaches and vestibular symptoms [27]. Migraines without aura present with recurrent, unilateral headaches that last between four hours and seventy-two hours. These headaches typically worsen with activity, and patients may describe the pain as throbbing with moderate to severe intensity [23]. For migraine with aura to be diagnosed, a patient would report at least two episodes that involve one or more aura symptoms, which including some unilateral visual, sensory, or other neurological symptoms, with visual aura being the most common [23]. These episodes also include gradual increase in the aura symptoms, development of unilateral aura symptoms, the duration of aura symptoms of under sixty minutes, or having any aura symptoms with a headache [23,29]. 

## 3. Current Treatment of Migraines

Migraine treatment consists of pharmacological, non-pharmacological, and neurostimulation treatment [32]. Non-pharmacological treatment can be further broken down into behavioral intervention. Pharmacological treatment will be discussed first. Treatments are broken down into preventive and abortive in Table 1.

### 3.1. Pharmacological Treatments

First-line pharmacological treatment for migraines are used for mild to moderate migraines when used alone, which include nonprescription non-steroidal anti-inflammatory drugs (NSAIDs) such as aspirin, acetaminophen products, and caffeine-containing combination analgesics [32,33,34]. These analgesics are first-line therapies because they are low-cost, effective, and practical since they are available over-the-counter [34]. Goldstein et al. found that a combination of acetaminophen/aspirin/caffeine treatment in cases of more severe migraines was more effective than 400 mg of ibuprofen [34]. Additionally, moderate to severe migraines and those that are mild to moderate but have not responded to analgesics can be treated with triptans, which are drugs specific for migraine treatment [34]. Triptans are a family of drugs that function by binding to serotonergic receptors [34]. There are seven different triptans currently used for treatment and they differ solely in their pharmacokinetics [34]. Triptans are 5-HT_1B/1D_ receptor agonists with some affinity for the 5-HT_1F_ [35]. Menstrual and hormone related migraines are a type of migraine that can be treated with triptans [36]. Perimenstrual eletriptan is a recommended drug for menstrual migraines which has been shown to cause a 46% decrease in headache activity [37]. Additionally, frovatriptan and naratriptan, which are long acting triptans, can be used to decrease the severity and frequency of menstrual migraines when used as short-term prevention [34]. 

Ditans are another class of medications, one of which that has been studied in lasmiditan. Ditans are specific 5-HT_1F_ agonist [35]. Two phase III trials, SAMURAI and SPARTAN, looked at the use of lasmiditan in patients using concomitant migraine preventive medications. Among those in the study, 17.5% were using preventative migraine treatment. All doses of lasmiditan in these studies resulted in significantly more patients being pain free at the two-hour mark [38]. The GLADIATOR study examined the long-term safety and efficacy of Lasmiditan. This study found that at 100 mg and 200 mg was shown to be generally well tolerated and efficacious for acute migraine treatment over a 1-year period [39]. 

In emergency departments, NSAIDs and intravenous (IV) antiemetics are an effective treatment that can be used with or without IV dihydroergotamine [34]. These drugs work in reducing immediate pain but are not necessarily used for treating long-term chronic migraines [32]. Opiates are not recommended for treatment of migraines due to dependency [32]. The discussed treatments are good for acute abortive therapy. The next sections look at possible preventive therapy of migraines.

### 3.2. Behavioral Interventions

A study by Goldstein et al. found that nonprescription medications were first used by more than fifty percent of patients in an attempt to treat their migraines before they visited a physician due to unsuccessful treatment [40]. Cognitive behavioral therapy (CBT) and relaxation training are nonpharmacological treatments associated with the prevention of migraines as well as an improvement of symptoms [32]. These treatments are recommended to be used alongside pharmacological treatment for chronic migraines [41,42]. Stress management is the traditional focus of CBT, while behavioral interventions, mindfulness, and meditation are directed towards treatment for comorbidities [41,42]. Relaxation techniques aim to lower arousal of the sympathetic system as well as lower muscle tone to promote relaxation of the body [41]. The focus of mindfulness therapies is to concentrate on sensations of the body [32]. Additionally, biofeedback is a self-regulatory behavioral intervention for migraine treatment with the purpose of helping patients acquire voluntary control of certain physiologic functions [43]. For biofeedback therapy, a healthcare professional monitors patients carefully as their physiologic functions are detected via surface sensors in real time, the results can be interpreted, and explain the process to the patient as they work [41,43]. For the best management of migraines, adoption of behavioral therapies alongside pharmacological treatment aids in treatment of symptoms as the avoidance of their onset [41].

### 3.3. Neurostimulation

Neurostimulation modalities have recently provided advantageous treatment opportunities for migraines as they are not limited by side effects the way pharmacological treatments are [32]. Non-invasive vagal nerve stimulation (nVNS), transcranial magnetic stimulation (TMS), occipital nerve stimulation (ONS), and supraorbital transcutaneous stimulation (SMS) therapies [32]. A handheld device is used to deliver nVNS, which inhibits vagal afferent fibers, while having no effect on vagal efferents that could result in bronchospasm and bradycardia [44]. A study by Silberstein et al. showed that some patients who received nVNS treatment had a reducation in migraine days by more than 50% [45]. ONS functions through peripheral and central mechanisms to reduce pain associated with migraines by lowering activation of certain brain regions [32]. TMS works by using a magnetic field to deliver a current to brain tissue which can cause functional activation or functional deactivation of areas of the brain [32]. This treatment was found to have favorable outcomes for patients who received treatment for migraines with aura [32]. Additionally, SMS, which is similar to ONS, is currently used for migraine prophylaxis throughout Europe as well as in the United States [32]. A study by Magis et al. found that patients who received SMS experienced a significant reduction in their number of headache days as compared to the control group [46].

### 3.4. Preventative Treatments

Prevention is a major focus in migraine treatment because it can be more effective in offsetting symptoms [32]. Some examples of effective classes of drugs in prevention are beta blockers, tricyclic antidepressants, anticonvulsants and botulinum derivatives [32,33,47]. Topiramate, an anticonvulsant, has a method of action involves minimizing excitatory effects as well as enhancing inhibitory effects, both of which work on neurotransmission [47]. The underlying anti-migraine mechanisms of topiramate’s activity potentially include carbonic anhydrase isozymes inhibition, neurotransmitter release modulation, and cell membrane ion channel regulation [47]. OnabotulinumtoxinA functions in preventing inflammatory neuropeptide release from trigeminal sensory neurons that have been stimulated [47]. These two preventative treatments have been reported to help decrease headache frequency, intensity of headaches, and number of doses required for symptomatic treatment [32]. Additionally, Cho, Song, & Chu found that botulinum toxin A (BT-A) can be effective in prevention of chronic migraines [32]. In patients receiving BT-A, occurrence of side effects and failure of treatment was lower than topiramate [32]. In regard to the prevention of episodic migraine, propranolol is regarded as the first-choice drug [33,48]. Also listed as other first line medications for the prevention of migraine include other beta blockers like metroprolol and timolol as well as divalproex [48]. Second line medications are amitriptyline, venlafaxine, atenolol, and nadolol as there is some evidence that they could be effective for prevention [48].

Anti-CGRP receptor monoclonal antibodies have been approved for the prevention of migraine by the FDA as well [21]. These monoclonal antibodies are thought to block the GCRP receptor and thereby downregulating the amount of glutamate which is released. These treatments are marketed under the names erenumab, galcanezumab, and fremanezumab [21]. A study that looked at the efficacy of erenumab showed that patients experienced fewer number of monthly migraine days than placebo [49]. The same findings were observed with galcanezumab and fremanezumab [50,51]. The draw back on these treatments is that they are self-administered injectables which can hinder the use in some patients. Common side effects noted were redness and swelling at the injection site as well as flu-like systems.

## 4. Urbrogepant

Urbrogepant is the first drug approved in the United States for the acute treatment of migraine with or without aura [22,52,53]. However, it is not indicated for use as a preventative treatment for migraines [22,54]. Ubrogepant is a highly selective, CGRP antagonist which blocks the vasodilatory action of CGRP and CGRP’s involvement in nociceptive transmission and modulation [22,52]. This medication can be equally as effective in treating migraine-associated symptoms which include nausea, photophobia, and sound sensitivity [54]. Ubrogepant shows a favorable safety profile, making it a promising treatment [55].

### 4.1. Usage

Ubrogepant is typically taken orally with a recommended dose of either 50 or 100 mg [52,53]. After at least two hours, a second dose may be taken, but there is a maximum dose of 200 mg per day [52,53]. The elimination half-life is approximately 5–7 h, and it is excreted mostly through fecal matter [53].

### 4.2. Cautions

If given in the presence of drugs that act as Cytochrome P450 3A4 (CYP3A4) inhibitors or inducers, ubrogepant concentration must be monitored, as those drugs will increase or decrease the amount of ubrogepant exposure, respectively [22,46]. Potent inhibitors of CYP3A4 include clarithromycin, erythromycin, diltiazem, itraconazole, ketoconazole, ritonavir, verapamil, goldenseal and grapefruit. Inducers of CYP3A4 include phenobarbital, phenytoin, rifampicin, St. John’s Wort and glucocorticoids.

Furthermore, the use of inhibitors for breast cancer resistance protein (BCRP) and P-glycoprotein (P-gp) may increase exposure to ubrogepant, so patients on any medications which might alter levels should be monitored to assess needed dosage changes [53]. BCRP inhibitors include anti-HIV protease inhibitors nelfinavir and ritonavir, the dietary flavonoids chrysin and biochanin A and P-gp inhibitors include amiodarone, clarithromycin, propafenone, and quinidine. Ubrogepant administration should be reduced to a 50 mg dose in patients with severe renal impairment or avoided altogether in patients with end stage renal disease as renal impairment will increase the patient’s exposure to the drug [53]. This medication should also be avoided in pregnant patients related to its ability to cause fetal harm [53].

## 5. Pharmacological Considerations

The sensation of migraine pain is thought to come from the activation of trigeminal ganglia in the nervous system [56,57]. These ganglia have afferent fibers that project to the spinal cord, synapse on various pain-sensing structures, and send signals of pain to auditory, visual, and motor cortices [56,57]. CGRP, which is abundant in these ganglia, is released from nerve terminals. It is also secreted within the trigeminal ganglia to perpetuate sensitization [56,58]. The sensitization is amplified by the interaction of CGRP with satellite glial cells and nearby neurons [58]. Trigeminal nerves potentiate the spread of inflammatory and neoplastic diseases, so the increased sensitivity of trigeminal nerves plays a role in the painful feeling experienced during migraine [55,58].

### Mechanism of Action

Ubrogepant has a high selectivity for CGRP receptors [59]. The exact sites for binding of CGRP in relation to migraine are still unknown [60]. By decreasing the amount of CGRP receptor binding, ubrogepant reduces the level of CGRP secreted into the trigeminal ganglia [56,58]. Thus, ubrogepant indirectly decreases the sensitivity of trigeminal nerves and reduces the amount of pain experienced [55,58]. Due to its ability to reduce pain in an effective way, this drug works well as an acute treatment of migraines [56].

Ubrogepant is part of a class of molecules called gepants [57]. At clinically effective exposures of ubrogepant, the drug does not cross the blood brain barrier [59]. Ubrogepant also has a fairly short half-life, good tolerability, and high potency, and it does not affect liver function. These properties make it a drug with a favorable safety profile [53,55,61,62].

## 6. Pharmacodynamics and Pharmacokinetics

Current treatments available to patients with migraines are limited by the non-specific nature of these treatments. Some of these therapies are not viable options for individuals with certain cardiovascular risk factors [63]. Ubrogepant is a possible treatment option for patients with chronic migraine, as it is more selective, specifically for CGRP receptors. It is safer for migraine patients with potential cardiovascular diseases as it does not prolong the QT interval [64]. Subtherapeutic and supratherapeutic doses (100–400 mg) had no effect on the QTc interval [64].

### 6.1. Pharmacodynamics

Ubrogepant has been shown to display antagonist activity of CGRP receptors with high selectivity when compared to similar medications in the same calcitonin receptor drug class [52,65]. Upon binding, ubrogepant blocks CGRP action to inhibit adenosine monophosphate to alpha-CGRP stimulated cyclic adenosine monophosphate responses [59]. Due to low saturation of CGRP receptors in the central nervous system, ubrogepant is known not to cross the blood brain barrier. While this does not explain the exact site of action of ubrogepant, this does confirm that it acts on non-central CGRP receptors, such as those in the trigeminal ganglia. Ubrogepant also shows some binding affinity for amylin receptors in trigeminal ganglia [59]. 

### 6.2. Pharmacokinetics

Ubrogepant is rapidly absorbed after oral administration and reaches its peak plasma concentration in roughly one and half hours [64,65]. Absorption of ubrogepant is affected by consumption of food, delaying the time to reach peak plasma concentration by two hours [64]. Metabolism of ubrogepant occurs primarily via CYP3A4 enzymes in the liver. The main metabolites produced are M15 and M20 glucuronide conjugates, which circulate in plasma [66]. The half-life of ubrogepant was found to be approximately 4 h following a 100 mg dose [65]. The main route of elimination is the biliary-fecal route while the minor route of elimination is renal [64].

## 7. Clinical Studies

### 7.1. Phase I Studies

A randomized, placebo-controlled, multicenter, double-blind trial was conducted over 8 weeks with an initial 4-week screening period and a follow up period for safety, also lasting 4-weeks [67]. The trial took place at 6 different centers across the United States starting in November 2017 and lasting until May 2018 [67]. Patients were randomized to receive two placebo tablets or to receive 100 mg dose of ubrogepant as 2 pills of 50 mg [67]. The treatment was administered to patients two times a week at the trial centers [67]. Patients’ blood samples were analyzed post-dose at either 0.5 h, 1 h, or 2 h [67]. Safety and tolerability were the primary outcomes measured [67]. The trial monitored treatment-emergent adverse events (TEAEs) which were defined as presenting after the first dose but also as adverse events (AEs) that previously presented but increased in severity after the first dose [67]. Hepatic safety was closely monitored, and serum chemistry values were taken at the two initial screening visits as well as on multiple days throughout the trial [67]. The TEAEs of most interest were elevated alanine aminotransferase (ALT) or aspartate aminotransferase (AST) levels and any suicidal behavior or ideation [67]. A total of 518 participants were involved in the trial. A total of 26 participants from the placebo and 26 of the participants from the ubrogepant group left the trial before completion, leaving 468 of the original 516 participants to finish the double-blinded treatment period lasting 8 weeks [67]. Ubrogepant and the placebo demonstrated similar overall incidence of TEAEs: ubrogepant (44%) vs. placebo (45%) [67]. For TEAEs, 89% of participants in both the placebo and ubrogepant groups experienced mild TEAEs while 11% of the placebo group and 8% of the ubrogepant ground experienced TEAEs of moderate severity [67]. The TEAEs reported the most frequently were oropharyngeal pain, nasopharyngitis and headache [67]. There were no participant deaths reported throughout the trial [67]. A participant in the ubrogepant group also experienced serious AEs associated with a car accident such as arthralgia, musculoskeletal pain, back pain, abdominal pain, and neck pain [67]. Of the patients who left the trial, three members of the placebo group left the trial due to dehydration, vomiting, influenza, pain in extremities, or tooth infection [67]. However, no patient in the ubrogepant group left due to AEs [67]. No patients reported TEAEs related to suicidal behaviors or ideation, as measure on the Columbia-Suicide Severity Rating Scale [67]. With regards to hepatic safety, only 3 members of the placebo group and 2 members of the ubrogepant group presented with increased ALT levels and 4 members of the placebo group and no members of the ubrogepant group presented with increased AST levels [67]. The trial identified no concerns with regards to safety associated with ubrogepant at high-frequency doses [67]. This trial demonstrated that ubrogepant at intermittent doses was not associated with elevation in levels of ALT or AST when compared to the placebo, it was not associated with the development of any liver injury deemed clinically significant [67]. 

### 7.2. Phase II Studies

A randomized computer-generated schedule was prepared in which participants were designated to a group in a double-blinded manner by a statistician [68]. There was a total of 834 participants randomized where 138 were assigned to 1 milligram of ubrogepant. 139 were assigned to 10 mg, 139 to 25 mg, 139 to 50 mg, 140 to 100 mg, and 139 to the placebo [68]. The trial took place in the United States at 55 study centers starting in July 2012 lasting until December 2012 [68]. The most frequent post-dose AEs were nausea, dizziness, fatigue, somnolence and dry-mouth, which presented within 48 h [68]. Within 14 days, the most frequent AEs were the same as those presenting within 48 h [68]. The placebo and ubrogepant groups had an incidence of AEs comparable to that associated with triptans, with neither group exhibiting any instances of asthenia, dysesthesia, hypesthesia, or chest or throat tightness [68]. There were no deaths or serious AEs within 14 days following the dose and no patients left the trial due to AEs [68]. Only two serious AEs were observed, both within the 50 mg group which were myoclonus occurring 15 days following treatment and hypertension [68]. The trial found that at higher doses, ubrogepant was beneficial for sustaining freedom from pain but also noted that further trials needed to be conducted for more definitive conclusions [68]. Ubrogepant demonstrated a statistically significant rate of success for freedom from pain within two hours that was higher than that of the placebo (25.5% vs. 8.9%, *p* = 0.003) throughout the dose range [68]. Additionally, both the 25 mg and 50 mg dose of ubrogepant demonstrated a pain-free two-hour rate than the placebo demonstrated (21% vs. 8.9%, *p* = 0.020 and 21.4% vs. 8.9%, *p* = 0.023 respectively) [68]. The 100 mg dose of ubrogepant showed a higher response rate to headaches compared to the placebo but the difference was not found to be statistically significant (58.8% vs. 44.6%, *p* = 0.061) [68]. Only the 25 mg, 50 mg, and 100 mg doses of ubrogepant demonstrated significant superiority over the placebo [68]. The effects of ubrogepant were comparable to those seen with telcagepant and triptans [68].

An additional trial was conducted to determine the safety, side effects, and efficacy of ubrogepant. This trial was double-blinded, randomized, and placebo-controlled with parallel groups [69]. Participants received either placebo, a 50 mg dose of ubrogepant, or a 100 mg dose of ubrogepant after being randomized into groups based on prior use of preventative migraine medication and prior response to triptans [69]. For the trial, participants reported their pain as either no pain, mild pain, moderate pain, or severe pain [69]. Additionally, they reported if various non-headache symptoms were absent or present as different times: prior to initial dose; at 0.5, 1, 1.5, 2, 3, 4, 6, 8, 14 and 48 h post dose, at the time of the second dose if they chose to do a second dose, and at two hours after the second dose [69]. Participants also stated the migraine symptom that bothered each of them most and reported its presence or absence at 2 h following each dose [69]. A primary efficacy analysis was conducted to assess the percentage of the participants who reported satisfaction with the treatment as well as the percentage who reported they were able to function normally [69]. The trial consisted of 1672 participants who were randomly assigned to 1 of 3 groups: 559 to placebo, 556 to 50 mg, and 557 to 100 mg [69]. The percentage of participants with freedom from pain 2 h following the initial dose was 11.8 of the placebo group, 19.2 of the 50 mg ubrogepant group (*p* = 0.002), and 21.2 of the 100 mg group (*p* < 0.001) [69]. Absence of the migraine associated symptom deemed most bothersome was reported in 27.8% of the placebo group, 38.6% of the 50 mg group (*p* = 0.002), and 37.7% of the 100 mg group (*p* = 0.003) [69]. Additionally, 336 participants of the 871 (38.6%) in the two ubrogepant groups choose to receive a second dose. Of those who took the second dose, 20.0% received ubrogepant [69]. The percentage of participants who experienced pain relief at the 2 h mark was 49.1% in the placebo group, 60.7% of the 50 mg ubrogepant group (*p* = 0.002), and 61.4% of the 100 mg (*p* = 0.002) [69]. At the 2 h mark, 29.8% of the placebo group reported no disability and were able to function as normal, 40.6% of the 50 mg group reported no disability and ability to function as normal, and 42.9% of the 100 mg group reported no disability and ability to function as normal [69]. AEs that presented or worsened within 48 h following the initial dose were reported in 12.9% of the participants, and 26.3% of the participants reported AEs within 30 days following any dose [69]. For this trial, the percentage of participants in the 50 mg dose or the 100 mg dose of ubrogepant were free from pain and the absence of other migraine-associated symptoms was higher among than those who received the placebo [69].

### 7.3. Phase III Trials

A randomized, multicenter, trial was conducted where patients were randomized to either placebo, 25 mg, or 50 mg of ubrogepant in a 1:1:1 ratio [70]. Randomization was based on past response to triptan treatment, and preventative migraine medication [70]. For the trial, participants took 1 tablet of their assigned medication as soon as possible within 4 h of noticing their symptoms [70]. To qualify for the trial, patients had to meet the following conditions: exhibit phonophobia or nausea, be able to self-administered medicine within the 4-h window of symptom onset, have migraine headaches falling into either moderate or severe category, have no usage of medicine that is prohibited (e.g., Ergot derivative, opioid, NSAIDs, antiemetic agents, analgesics, proton pump inhibitors, or triptans), exhibit symptoms of a new migraine, and have had a migraine that was not previously resolving [70]. At two hours after the initial dose, both the absence of migraine-associated symptom considered most bothersome and pain were evaluated [70]. Freedom from pain was considered to be a reduction in headache severity from baseline of moderate to severe pain to no pain [70]. Participants completed the Functional Disability Scale before dosing as well as at 1, 2, 4, and 8 h post original dose [70]. Patients responses ranged from 0 which was ability to function at normal capacity to 3 which was considered to be impaired in serious manner, cannot perform at their normal level, and may require bed rest [70]. For the 50 mg group, patients reported freedom from pain at the 2-h mark was significantly greater compared to the placebo (21.8% vs. 14.3%, *p* = 0.01) and the 25 mg group was also found to be significantly greater compared to the placebo (20.7% vs. 14.3%, *p* = 0.03) [70]. For patients reporting absence of the migraine-associated symptom considered most bothersome at the 2 h mark, the percentage was significantly greater for the 50 mg dose (38.9% vs. 27.4%, *p* = 0.01) compared to the placebo but was not significantly greater for the 25 mg dose (34.1% vs. 27.4%, *p* = 0.07) [70]. All clinical studies are summarized below in Table 2.

## 8. Conclusions

Affecting over 16% of the entire United States population and approximately 1–2% of the population around the world, migraine is a condition that impairs the daily activities of many individuals [2,25]. Therefore, it is important to find a treatment that can reduce its painful symptoms in order to help patients return to their normal everyday lives. Migraines can be treated in variety of different ways. Some patients may prefer the pharmacological route while others may prefer the behavioral one. For avoidance of medication, cognitive behavioral therapy, relaxation therapy, or nerve stimulation may be ideal routes for treatment. On the other hand, if medication is preferred, there are a few types that may be considered and some of them may be more effective than others. NSAIDS, triptans, and some analgesics are known for the treatment of migraine, while topiramate, propranolol, amitriptyline, divalproex, verapamil and OnabotulinumtoxinA are examples of drugs used for preventative treatment [32,33,47]. Triptans and ditans are similar as they both agonize a 5-HT receptor, however, ditans such as lasmiditan are specific 5-HT_F1_ agonists. Both triptans and ditans need to be taken within two hours of the start of a migraine. Triptans are mostly nonspecific in terms of their receptor actions. Ubrogepant, by contrast, is a specific CGPR antagonist. In addition, the emerging medication ubrogepant, which has been very recently approved for the acute treatment of migraine with or without aura, has been shown to be an effective and safe therapeutic agent. As of this writing, ditans are not approved for use in the European Union, however, gepants such as ubrogepant are approved [52].

As a CGPR antagonist, ubrogepant works to block CGRP release at locations within the migraine pathway [3,21]. This drug’s high specificity and selectivity for CGRP sets it apart from certain other drugs, which previously limited the treatment of migraines with or without aura due to their decreased selectivity [53,59]. There are a few important cautions to ubrogepant which are to avoid taking it when pregnant or with end stage renal disease [53]. However, overall, it has good tolerability and an overall favorable safety profile [55]. In conclusion, it appears to hold promise for the acute treatment of migraines with or without aura in adults.

## Figures and Tables

**Table 1:** Current Treatments in Migraines.

Preventative Treatment	Abortive Treatment
CBT Relaxation training Biofeedback Anticonvulsants (i.e., topiramate) OnabotulinumtoxinA BT-A Beta Blockers (i.e., propranolol, metroprolol and timolol) TCAs (i.e., mitriptyline) Venlafaxine Neurostimulation: nVNS, TMS, ONS, SMS therapies Anti-CGRP monoclonal antibodies	non-steroidal anti-inflammatory drugs Triptans (i.e., eletriptan, sumatriptan, frovatriptan and naratriptan) IV dihydroergotamine CGRP antagonists (i.e., ubrogepant)

**Table 2:** Clinical Efficacy and Safety—Ubrogepant.

Author (Year)	Groups Studied and Intervention	Results and Findings	Conclusions
Goadsby, P.J., et al., (2019)	Healthy males or females between the ages of 18 and 50 years Participants were required to have normal ALT and AST levels and were excluded if they had any of the following: sitting systolic blood pressure ≥140 or ≤90 mm Hg or diastolic blood pressure ≥90 or ≤50 mm Hg, history of clinically significant reaction or hypersensitivity to CGRP.	For TEAEs, 89% of participants in both the placebo and ubrogepant groups experienced mild TEAEs while 11% of the placebo group and 8% of the ubrogepant ground experienced TEAEs of moderate severity. The TEAEs reported the most frequently were oropharyngeal pain, nasopharyngitis and headache.	The trial identified no concerns in regard to safety associated with ubrogepant at high-frequency doses.
Voss, T., et al., (2016)	Participants ranged from 18–65 years of with a ≥1-year history of migraines who experienced between two and 8 moderate or severe migraine attacks every month for at least two months prior to trial screening.	Ubrogepant demonstrated a statistically significant rate of success for freedom from pain within two hours that was higher than that of the placebo (25.5% vs. 8.9%, *p* = 0.003). Additionally, 50 mg dose of ubrogepant demonstrated a nominally significantly (*p* value < 0.05 unadjusted) higher pain-free two-hour rate than the placebo demonstrated (21% vs. 8.9%, *p* = 0.020). The 100 mg dose of ubrogepant showed a higher response rate to headaches compared to the placebo but the difference was not found to be statistically significant (58.8% vs. 44.6%, *p* = 0.061).	Demonstrated a positive response trend, measured as the proportion of participants achieving freedom from pain after 2 h.
Dodick, D.W., et al., (2019)	Participants were between the ages of 18 and 75 years old with at least ≥1-year of history of migraine and had onset of migraines before the age of 50. Participants had to have a history of migraines lasting between 4 to 72 h in length and migraines separated by at least 48 h pain-free. Participants needed to have a history of at least 2 to 8 migraines per month that were rated as moderate or severe with onset before 3 months of the start of the trial.	The number of participants with pan-freedom 2 h following the initial dose was 11.8% of the placebo group, 19.2% of the 50 mg ubrogepant group (*p* = 0.002), and 21.2% of the 100 mg ubrogepant group (*p* < 0.001).	The percentage of participants in the 50 mg dose or the 100 mg dose of ubrogepant had pain-freedom ad absence of migraine-associated symptoms deemed most bothersome.
Lipton, R.B., et al., (2019)	Participants were between the ages of 18 and 75 years old with a history of migraine for at least 1 year and experienced 2 to 8 migraine attacks rated moderate to severe presenting each month for 3 months before the screening for the trial. Participants were required to have migraine onset before the age of 50 with 48 h of freedom from pain between migraines.	For the 50 mg dose of ubrogepant, patients reported freedom from pain at the 2-h mark was significantly greater compared to the placebo (21.8% vs. 14.3%, *p* = 0.01). The 25 mg dose was also found to be significantly greater compared to placebo (20.7% vs. 14.3%, *p* = 0.03).	Ubrogepant at doses of 50 mg and 25 mg was found to produce greater rates of freedom from pain after 2 h compared to the placebo [70].

## Data Availability

All supporting data was discussed in this review.

## Abbreviations

CGRP	Calcitonin gene-related peptide
5-HT	5-hydroxytryptamine
PACAP	Pituitary adenylate cyclase activating polypeptide
NSAIDs	non-steroidal anti-inflammatory drugs
IV	intravenous
CBT	Cognitive Behavioral Therapy
nVNS	Non-invasive vagal nerve stimulation
TMS	transcranial magnetic stimulation
ONS	occipital nerve stimulation
SMS	supraorbital transcutaneous stimulation
BT-A	botulinum toxin A
CYP3A4	Cytochrome P450 3A4
BCRP	breast cancer resistance protein
P-glycoprotein	P-gp
TEAEs	treatment-emergent adverse events
AEs	adverse events
ALT	alanine aminotransferase
AST	aspartate aminotransferase

## References

[1] Silberstein S.D. (2004). Migraine. Lancet.

[2] MacGregor E.A. (2017). Migraine. Ann. Intern. Med..

[3] Migraine Overview and Summary of Current and Emerging Treatment Options|AJMC. https://www.ajmc.com/view/migraine-overview-and-summary--of-current-and-emerging-treatment-options.

[4] Cologno D., Mazzeo A., Lecce B., Mundi C., Petretta V., Casucci G., d’Onofrio F. (2012). Triptans: Over the migraine. Neurol. Sci..

[5] Charles A. (2018). The pathophysiology of migraine: Implications for clinical management. Lancet Neurol..

[6] Dodick D.W. (2018). A Phase-by-Phase Review of Migraine Pathophysiology. Headache.

[7] Charles A. (2013). The evolution of a migraine attack—A review of recent evidence. Headache.

[8] Olesen J., Friberg L., Olsen T.S., Iversen H.K., Lassen N.A., Andersen A.R., Karle A. (1990). Timing and topography of cerebral blood flow, aura, and headache during migraine attacks. Ann. Neurol..

[9] Minen M.T., De Dhaem O.B., Van Diest A.K., Powers S., Schwedt T.J., Lipton R., Silbersweig D. (2016). Migraine and its psychiatric comorbidities. J. Neurol. Neurosurg. Psychiatry.

[10] Merikangas K.R., Angst J., Isler H. (1990). Migraine and Psychopathology: Results of the Zurich Cohort Study of Young Adults. Arch. Gen. Psychiatry.

[11] Merikangas K.R., Merikangas J.R., Angst J. (1993). Headache syndromes and psychiatric disorders: Association and familial transmission. J. Psychiatr. Res..

[12] Breslau N., Davis G.C., Andreski P. (1991). Migraine, psychiatric disorders, and suicide attempts: An epidemiologic study of young adults. Psychiatry Res..

[13] Rains J.C., Poceta J.S. (2006). Headache and sleep disorders: Review and clinical implications for headache management. Headache.

[14] Lipton R.B., Hamelsky S.W., Kolodner K.B., Steiner T.J., Stewart W.F. (2000). Migraine, quality of life, and depression: A population-based case-control study. Neurology.

[15] Zwart J.A., Dyb G., Hagen K., Ødegård K.J., Dahl A.A., Bovim G., Stovner L.J. (2003). Depression and anxiety disorders associated with headache frequency. The Nord-Trøndelag Health Study. Eur. J. Neurol..

[16] Tietjen G.E., Peterlin B.L. (2011). Childhood abuse and migraine: Epidemiology, sex differences, and potential mechanisms. Headache.

[17] Schwedt T.J. (2014). Chronic migraine. BMJ.

[18] Su M., Yu S. (2018). Chronic migraine: A process of dysmodulation and sensitization. Mol. Pain.

[19] Strupp M., Versino M., Brandt T. (2010). Vestibular migraine. Handb. Clin. Neurol..

[20] Von Brevern M., Lempert T. (2016). Vestibular migraine. Handbook of Clinical Neurology.

[21] Tepper S.J. (2018). History and Review of anti-Calcitonin Gene-Related Peptide (CGRP) Therapies: From Translational Research to Treatment. Headache.

[22] Ubrelvy (Ubrogepant) Dosing, Indications, Interactions, Adverse Effects, and More. https://reference.medscape.com/drug/ubrelvy-ubrogepant-1000348.

[23] Olesen J. (2018). Headache Classification Committee of the International Headache Society (IHS) The International Classification of Headache Disorders, 3rd edition. Cephalalgia.

[24] May A., Schulte L.H. (2016). Chronic migraine: Risk factors, mechanisms and treatment. Nat. Rev. Neurol..

[25] Burch R.C., Buse D.C., Lipton R.B. (2019). Migraine: Epidemiology, Burden, and Comorbidity. Neurol. Clin..

[26] Diener H.C., Solbach K., Holle D., Gaul C. (2015). Integrated care for chronic migraine patients: Epidemiology, burden, diagnosis and treatment options. Clin. Med. J. R. Coll. Physicians Lond..

[27] Furman J.M., Marcus D.A., Balaban C.D. (2013). Vestibular migraine: Clinical aspects and pathophysiology. Lancet Neurol..

[28] Lempert T., Olesen J., Furman J., Waterston J., Seemungal B., Carey J., Bisdorff A., Versino M., Evers S., Newman-Toker D. (2012). Vestibular migraine: Diagnostic criteria. J. Vestib. Res. Equilib. Orientat..

[29] Vgontzas A., Burch R. (2018). Episodic Migraine With and Without Aura: Key Differences and Implications for Pathophysiology, Management, and Assessing Risks. Curr. Pain Headache Rep..

[30] Todd C., Lagman-Bartolome A.M., Lay C. (2018). Women and Migraine: The Role of Hormones. Curr. Neurol. Neurosci. Rep..

[31] Buse D.C., Manack A., Serrano D., Turkel C., Lipton R.B. (2010). Sociodemographic and comorbidity profiles of chronic migraine and episodic migraine sufferers. J. Neurol. Neurosurg. Psychiatry.

[32] Cho S.J., Song T.J., Chu M.K. (2017). Treatment Update of Chronic Migraine. Curr. Pain Headache Rep..

[33] Huang T.C., Lai T.H., Wang S.J., Wang P.J., Wang Y.F., Lee L.H. (2017). Medical treatment guidelines for preventive treatment of migraine. Acta Neurol. Taiwanica.

[34] Gilmore B., Michael M. (2011). Treatment of acute migraine headache. Am. Fam. Physician.

[35] Do T.P., Guo S., Ashina M. (2019). Therapeutic novelties in migraine: New drugs, new hope?. J. Headache Pain.

[36] Warnock J.K., Cohen L.J., Blumenthal H., Hammond J.E. (2017). Hormone-Related Migraine Headaches and Mood Disorders: Treatment with Estrogen Stabilization. Pharmacotherapy.

[37] Nierenburg H.D.C., Ailani J., Malloy M., Siavoshi S., Hu N.N., Yusuf N. (2015). Systematic Review of Preventive and Acute Treatment of Menstrual Migraine. Headache.

[38] Loo L.S., Ailani J., Schim J., Baygani S., Hundemer H.P., Port M., Krege J.H. (2019). Efficacy and safety of lasmiditan in patients using concomitant migraine preventive medications: Findings from SAMURAI and SPARTAN, two randomized phase 3 trials. J. Headache Pain.

[39] Brandes J.L., Klise S., Krege J.H., Case M., Khanna R., Vasudeva R., Raskin J., Pearlman E.M., Kudrow D. (2019). Interim results of a prospective, randomized, open-label, Phase 3 study of the long-term safety and efficacy of lasmiditan for acute treatment of migraine (the GLADIATOR study). Cephalalgia.

[40] Goldstein J., Silberstein S.D., Saper J.R., Ryan R.E., Lipton R.B. (2006). Acetaminophen, aspirin, and caffeine in combination versus ibuprofen for acute migraine: Results from a multicenter, double-blind, randomized, parallel-group, single-dose, placebo-controlled study. Headache.

[41] Pistoia F., Sacco S., Carolei A. (2013). Behavioral therapy for chronic migraine. Curr. Pain Headache Rep..

[42] Starling A.J., Dodick D.W. (2015). Best Practices for Patients with Chronic Migraine: Burden, Diagnosis, and Management in Primary Care. Mayo Clin. Proc..

[43] Pérez-Muñoz A., Buse D.C., Andrasik F. (2019). Behavioral Interventions for Migraine. Neurol. Clin..

[44] Tepper S.J. (2019). Acute Treatment of Migraine. Neurol. Clin..

[45] Silberstein S.D., Calhoun A.H., Lipton R.B., Grosberg B.M., Cady R.K., Dorlas S., Simmons K.A., Mullin C., Liebler E.J., Goadsby P.J. (2016). Chronic migraine prevention with nVNS: The EVENT study. Neurology.

[46] Magis D., Sava S., d’Elia T.S., Baschi R., Schoenen J. (2013). Safety and patients’ satisfaction of transcutaneous supraorbital neurostimulation (tSNS) with the Cefaly^®^ device in headache treatment: A survey of 2,313 headache sufferers in the general population. J. Headache Pain.

[47] Aurora S.K., Brin M.F. (2017). Chronic Migraine: An Update on Physiology, Imaging, and the Mechanism of Action of Two Available Pharmacologic Therapies. Headache.

[48] Ha H., Gonzalez A., Rosa S. (2019). Migraine Headache Prophylaxis. Am. Fam. Physician.

[49] Tepper S., Ashina M., Reuter U., Brandes J.L., Doležil D., Silberstein S., Winner P., Leonardi D., Mikol D., Lenz R. (2017). Safety and efficacy of erenumab for preventive treatment of chronic migraine: A randomised, double-blind, placebo-controlled phase 2 trial. Lancet Neurol..

[50] Mulleners W.M., Kim B.K., Láinez M.J.A., Lanteri-Minet M., Pozo-Rosich P., Wang S., Tockhorn-Heidenreich A., Aurora S.K., Nichols R.M., Yunes-Medina L. (2020). Safety and efficacy of galcanezumab in patients for whom previous migraine preventive medication from two to four categories had failed (CONQUER): A multicentre, randomised, double-blind, placebo-controlled, phase 3b trial. Lancet Neurol..

[51] Ferrari M.D., Diener H.C., Ning X., Galic M., Cohen J.M., Yang R., Mueller M., Ahn A.H., Schwartz Y.C., Grozinski-Wolff M. (2019). Fremanezumab versus placebo for migraine prevention in patients with documented failure to up to four migraine preventive medication classes (FOCUS): A randomised, double-blind, placebo-controlled, phase 3b trial. Lancet.

[52] Scott L.J. (2020). Ubrogepant: First Approval. Drugs.

[53] Center for Drug Evaluation and Research, FDA Ubrogepant FDA. www.fda.gov/medwatch.

[54] Dhir A. (2020). Ubrogepant to treat migraine. Drugs Today.

[55] Curto M., Capi M., Cipolla F., Cisale G.Y., Martelletti P., Lionetto L. (2020). Ubrogepant for the treatment of migraine. Expert Opin. Pharmacother..

[56] Ubrogepant—DrugBank. https://go.drugbank.com/drugs/DB15328.

[57] Negro A., Martelletti P. (2019). Gepants for the treatment of migraine. Expert Opin. Investig. Drugs.

[58] Iyengar S., Johnson K.W., Ossipov M.H., Aurora S.K. (2019). CGRP and the Trigeminal System in Migraine. Headache.

[59] Moore E., Fraley M.E., Bell I.M., Burgey C.S., White R.B., Li C.C., Regan C.P., Danziger A., Michener M.S., Hostetler E. (2020). Characterization of ubrogepant: A potent and selective antagonist of the human calcitonin gene-related peptide receptors. J. Pharmacol. Exp. Ther..

[60] Wattiez A.S., Wang M., Russo A.F. (2019). CGRP in animal models of migraine. Handbook of Experimental Pharmacology.

[61] Allergan Enters into Licensing Agreement with Merck to Obtain Exclusive Worldwide Rights to CGRP Migraine Development Program. https://www.prnewswire.com/news-releases/allergan-enters-into-licensing-agreement-with-merck-to-obtain-exclusive-worldwide-rights-to-cgrp-migraine-development-program-300109311.html.

[62] De Matteis E., Guglielmetti M., Ornello R., Spuntarelli V., Martelletti P., Sacco S. (2020). Targeting CGRP for migraine treatment: Mechanisms, antibodies, small molecules, perspectives. Expert Rev. Neurother..

[63] Moreno-Ajona D., Chan C., Villar-Martínez M.D., Goadsby P.J. (2019). Targeting CGRP and 5-HT1F Receptors for the Acute Therapy of Migraine: A Literature Review. Headache.

[64] Allergan I. (2019). Ubrelvy.

[65] Jakate A., Boinpally R., Butler M., Lu K., McGeeney D., Periclou A. (2020). Single Therapeutic and Supratherapeutic Doses of Ubrogepant Do Not Affect Cardiac Repolarization in Healthy Adults: Results From a Randomized Trial. Clin. Pharmacol. Ther..

[66] Blumenfeld A.M., Edvinsson L., Jakate A., Banerjee P. (2020). Pharmacology and Pharmacokinetics of Ubrogepant: A Potent, Selective Calcitonin Gene-Related Peptide Receptor Antagonist for the Acute Treatment of Migraine. J. Fam. Pract..

[67] Goadsby P.J., Tepper S.J., Watkins P.B., Ayele G., Miceli R., Butler M., Severt L., Finnegan M., Szegedi A., Trugman J.M. (2019). Safety and tolerability of ubrogepant following intermittent, high-frequency dosing: Randomized, placebo-controlled trial in healthy adults. Cephalalgia.

[68] Voss T., Lipton R.B., Dodick D.W., Dupre N., Ge J.Y., Bachman R., Assaid C., Aurora S.K., Michelson D. (2016). A phase IIb randomized, double-blind, placebo-controlled trial of ubrogepant for the acute treatment of migraine. Cephalalgia.

[69] Dodick D.W., Lipton R.B., Ailani J., Lu K., Finnegan M., Trugman J.M., Szegedi A. (2019). Ubrogepant for the treatment of migraine. N. Engl. J. Med..

[70] Lipton R.B., Dodick D.W., Ailani J., Lu K., Finnegan M., Szegedi A., Trugman J.M. (2019). Effect of ubrogepant vs placebo on pain and the most bothersome associated symptom in the acute treatment of migraine: The achieve ii randomized clinical trial. JAMA J. Am. Med. Assoc..

